# Immune checkpoint blockade can synergize with radiation therapy, even in tumors resistant to checkpoint monotherapy

**DOI:** 10.15252/emmm.201607219

**Published:** 2016-12-13

**Authors:** Jan Dörrie

**Affiliations:** ^1^ Department of DermatologyUniversitätsklinikum ErlangenErlangenGermany

**Keywords:** Cancer, Digestive System, Immunology

## Abstract

Immunotherapy has evolved as a new pillar of cancer treatment during the last decade. The main breakthrough was the development of immune checkpoint blocking (ICB) antibodies, which antagonize inhibitory receptors on T cells and their ligands and thus unleash the cellular immune system against the tumor. ICB showed tremendous effects in several types of cancer. However, only a proportion of the patients suffering from tumors, which are in principle sensitive, benefit from this treatment and other kinds of neoplasia are completely resistant. Great effort is currently being undertaken to distinguish responders from non‐responders, and concepts to turn the latter into the former are urgently required. One approach is to combine ICB with already well‐established treatment strategies, that is, the other mainstays of cancer therapy such as surgery, radiation therapy (RT), and chemotherapy. Depending on the circumstances, both chemotherapy and RT may act either immune suppressively or immune stimulatingly. In this issue of *EMBO Molecular Medicine*, Azad *et al* (2017) show that indeed, pancreatic ductal adenocarcinoma, which is resistant to ICB monotherapy, becomes responsive to this treatment by simultaneous RT or chemotherapy.

Over the last decade, it has become clear that our immune system features very powerful control mechanisms required to keep a possibly self‐destructive system at bay, thus avoiding autoimmunity. Tumors hijack these mechanisms to evade immune response, and blocking these so‐called immune checkpoints (i.e. suppressive receptors expressed by T cells, namely CTLA‐4 and PD‐1) with therapeutic antibodies generated impressive results in a variety of tumors. These include malignant melanoma, Hodgkin lymphoma, Merkel cell carcinoma, and head and neck, breast, lung, gastric, hepatocellular, renal cell, ovarian, and colorectal cancer (reviewed by Callahan *et al*, 2016). However, only a small proportion of the patients suffering from these malignancies actually benefit from the treatment and several other malignancies respond poorly or are completely resistant.

One aggressive and usually fatal tumor that appears to be completely refractory to ICB is pancreatic ductal adenocarcinoma (PDAC; Royal *et al*, 2010; Brahmer *et al*, 2012). It is the fourth most common cause of cancer‐related death and has a 5‐year relative survival of only 7% (Siegel *et al*, 2015). Treatment options are limited and chemotherapy with gemcitabine is performed as standard treatment monotherapy. The combination with other chemotherapeutic agents increased overall survival but at the cost of massive side effects (Ellenrieder *et al*, 2016). Stereotactic body radiotherapy (SBRT) can provide solid local control of PDAC (Kim *et al*, 2016). The report by Azad *et al* (2017) provides first evidence in a murine model of PDAC that a combinational treatment of RT and ICB can provide better tumor control.

As mentioned above, the molecular basis underlying why only some tumors respond to ICB is currently under intense investigation. Neoepitopes generated by somatic mutations are thought to make the tumor visible for T cells, resulting in an increased probability that ICB will work. Therefore, the correlation of responsiveness with mutational load is generally accepted, but it is not strong enough to be of predictive value, and other factors must play a role as well (Hugo *et al*, 2016). Apart from the genetic alterations, changes of the transcriptional program in the tumors seem to be just as relevant (Hugo *et al*, 2016).

The use of ionizing radiation is one of the historical pillars of cancer therapy. It primarily affects rapidly dividing cells by damaging DNA. Next to tumor cells, leukocytes and their precursors are therefore highly sensitive to irradiation. Cancer RT was originally observed to be immune‐compromising, but that was mainly caused by off‐target damage to lymphatic tissue due to the unfocussed large‐area delivery of the radiation. Now irradiation can be precisely focused on the tumor, minimizing the effects on healthy tissue.

Under such conditions, a few patients showed tumor regression also outside the irradiated area. These immunologically mediated results, termed abscopal effects, indicate the induction of an adaptive cellular immune response against the cancer. Inside the tumor, radiation was shown to have immunomodulatory effects, which seem to facilitate a CTL response. The MHC and peptide repertoire are rapidly upregulated by RT (Reits *et al*, 2006). This effect is not caused by the induced mutations, but probably due to RT‐mediated protein damage, involving mTOR. The new epitopes were also shown to act as potential T‐cell targets (Reits *et al*, 2006). High‐mobility group protein B1 (HMGB1) and calreticulin seem to be involved as well, and immunoproteasome subunits are upregulated. The plethora of mostly pro‐inflammatory and CTL‐activating effects is accompanied by an induction of PD‐L1, counteracting the inflammation (Sharabi *et al*, 2015). Hence, the combination of RT with ICB is the next logical step (Derer *et al*, 2016). Although evidence of synergistic effects has been provided on a variety of preclinical murine tumor models in the last 2 years, these were mainly focused on an enhancement of ICB activity in already sensitive malignancies. The report of Azad *et al* (2017) stands out because it shows that a completely resistant tumor type is rendered sensitive to ICB under concurrent RT or chemotherapy. They observed that both RT and chemotherapy on the one hand upregulated PD‐L1 in a JAK/Stat‐dependent manner, while on the other, the intratumoral milieu was shifted away from immune suppressive myeloid cell infiltration and toward an activated CD8^+^ T‐cell signature. While anti‐PD‐L1 alone had no effect on the milieu, the simultaneous combination clearly synergized. The observation that sequential treatment had no effect argues against RT‐mediated mutagenesis as a key factor for ICB sensitivity in this system, although this requires further investigation. But despite the still incomplete knowledge of the molecular mechanisms involved, the central message of the publication of Azad *et al* (2017) clearly opens up new avenues for the large group of ICB‐resistant tumors, which all could benefit from the combinations.

Clinical research on combined treatments is rapidly progressing, and although few results have been published, about 40 clinical trials are already listed on ClinicalTrials.gov that examine concurrent treatment with PD‐1‐ or PD‐L1‐blocking antibodies and RT of patients suffering from glioblastoma, urothelial cancer, small‐ and non‐small‐cell lung cancer, Merkel cell carcinoma, head and neck cancer, metastatic melanoma, esophageal cancer, renal cell carcinoma, and colorectal cancer. Interestingly, unresectable non‐metastatic pancreatic cancer is also being investigated in a phase I trial (Status Nov. 2016: not yet recruiting, NCT02868632). The outcome of this trial will put the findings of Azad *et al* (2017) to the test in the clinical setting.

Clearly, the take‐home message, which was already well received in the field, is to hit the tumor simultaneously with as many different weapons as possible. The older strategy was overcome, to treat sequentially with first‐, second‐, and third‐line treatment while waiting each time until resistance occurred, and only after that try immunotherapy or other experimental treatments. The combination radiation and ICB treatment brings new hope, especially for those malignancies for which the current standard of care is not significantly efficacious.
